# A Case Report of Cystic Fibrosis Complicated by Burkholderia Cepacia and Cutaneous Vasculitis

**DOI:** 10.7759/cureus.8158

**Published:** 2020-05-16

**Authors:** Artsiom Klimko, Alienor Brandt, Maria-Iulia Brustan, Mihaela Balgradean

**Affiliations:** 1 Division of Physiology and Neuroscience, Carol Davila University of Medicine and Pharmacy, Bucharest, ROU; 2 Pediatrics, Carol Davila University of Medicine and Pharmacy, Bucharest, ROU; 3 Pediatrics Section, Marie Curie Children's Emergency Hospital, Bucharest, ROU; 4 Pediatrics Section, Marie Curie Children's Emergency Hospital, Bucharest , ROU

**Keywords:** cystic fibrosis, cutaneous vasculitis, burkholderia cepacia complex

## Abstract

While the pulmonary and pancreatic involvement of cystic fibrosis (CF) is commonly described and therefore best studied, the cutaneous manifestations are frequently underdiagnosed, despite being important markers of disease severity. We report a case of antineutrophil cytoplasmic antibody-negative cutaneous vasculitis in a 15-year-old female CF patient in tandem with infection and subsequent colonization by Burkholderia cepacia complex (BCC). The flares of cutaneous vasculitis is associated closely with an infective exacerbation of CF and improved upon treatment of the infective exacerbation. We further discuss how the appearance of BCC colonization and cutaneous vasculitis affected both lung function and lung parenchyma by tracking spirometry and imaging changes over the subsequent four years.

## Introduction

Cutaneous vasculitis is an unusual but well-established complication of cystic fibrosis (CF) that may serve as a predictor of severe lung disease [1]. It affects 2% to 3% of CF patients, although skin lesions are underestimated and, therefore, underdiagnosed [2]. However, it is crucial to recognize CF-related vasculitis as although is usually cutaneous, the vasculitis may become disseminated [1,3]. This necessitates prompt management with steroids and immunosuppressive treatment, as it may become systemic and rapidly fatal [1,4]. To date, there is only one study, comprised of four patients, which established and explored the association between reactive skin presentations and chronic Burkholderia cepacia complex (BCC) infections in CF patients [5]. However, the patients were evaluated retrospectively and there is no data that would allow to quantify lung function or evaluate structural lung changes prospectively. Furthermore, our case is also quite interesting from an academic point of view, as the antineutrophil cytoplasmic antibody (ANCA)-negative vasculitis was also associated with infective exacerbations of CF (IECF). Finally, the patient was also seropositive for rheumatoid factor and reported flares of arthritis, which overlapped with the appearance of the vasculitis. 

## Case presentation

**Figure 1:**
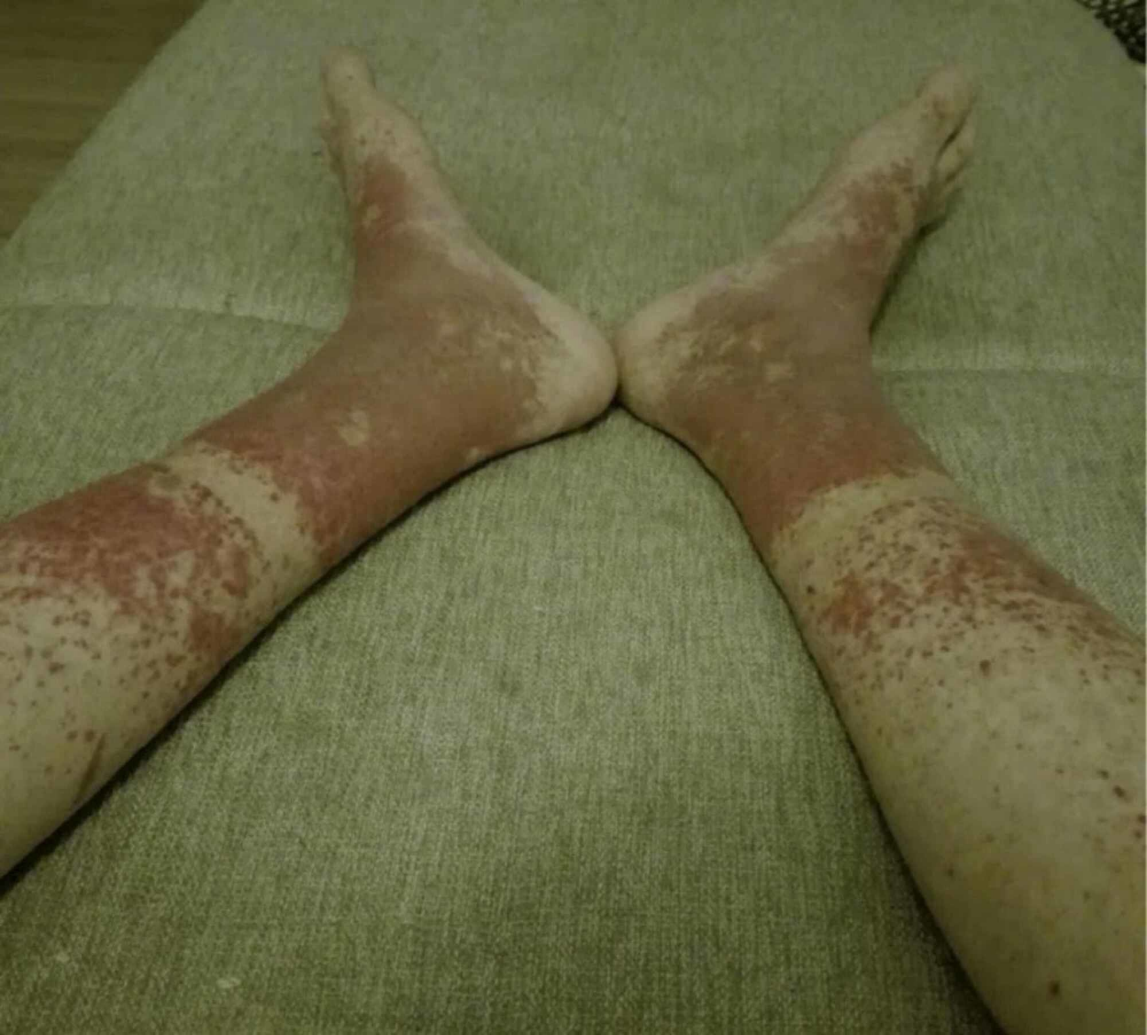
Clinical appearance of the legs of the patient upon admission (2015) showing a palpable purpuric rash

**Figure 2:**
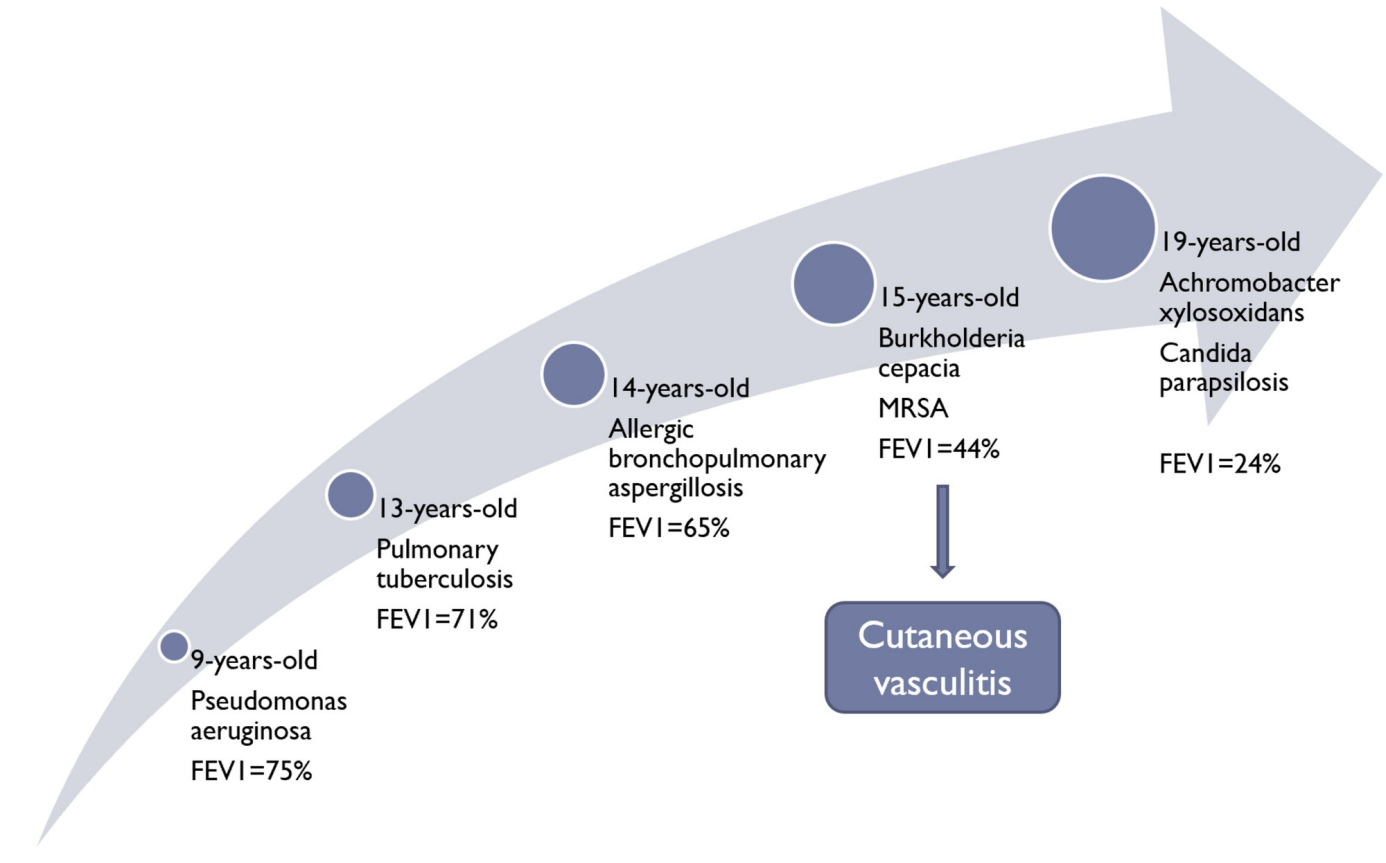
Summary of bacterial colonization and progression of respiratory function FEV1, forced expiratory volume in one second; MRSA, methicillin-resistant Staphylococcus aureus

We present the case of a 15-year-old female heterozygous for ΔF508del/N1303K, with poorly controlled lung disease determined by forced expiratory volume in one second (FEV1) 44% of predicted value, who presented with cutaneous vasculitis and arthritis in association with an infective exacerbation of CF. The cutaneous involvement consisted of a palpable purpuric rash distributed over the tibial surfaces, ankles, and dorsa of the feet (Figure 1). At this admission in 2015, infection and colonization with BCC and methicillin-resistant Staphylococcus aureus (MRSA) later in the year were also diagnosed. Blood work at admission is presented in Table 1; acute phase reactants (C-reactive protein and fibrinogen) and rheumatoid factor were elevated. ANCAs were negative. Antinuclear antibodies (ANAs) were negative initially, but became positive five years later. Over the next several years, the patient required on average three to four hospitalizations every year due to IECF. Cutaneous vasculitis was present in approximately every other episode of IECF and improved with treatment of the pulmonary exacerbations. Bacterial colonization together with respiratory function is summarized in Figure 2 - of note, the FEV1 decreased from 65% at 14 years of age (before appearance of the vasculitis) to 24% by 19 years of age.

**Table 1:** Relevant blood analyses upon admission (2015) Albumin, IgM levels, ferritin, IgE were all normal and there was no evidence of renal involvement. ANCA, antineutrophil cytoplasmic antibody; ANA, antinuclear antibody; IgA, immunoglobulin A; IgG, immunoglobulin G

Laboratory parameter	Patient laboratory values at presentation	Reference range
C-reactive protein (nmol/L)	32	103
Fibrinogen (mg/dL)	413	<374
IgA (mg/dL)	694	<348
IgG (mg/dL)	2067	<1584
ANCAs	Negative	-
ANAs	Negative (initially)	-
Rheumatoid factor	Positive	-

From the patient’s medical history, we consider eloquent the following facts: at the age of two years, she received nine-month isoniazid prophylaxis due to contact with her father who had tuberculosis. At the age of three years, she was diagnosed with bronchiectasis via CT. At the age of nine years, colonization with Pseudomonas aeruginosa was diagnosed through bronchoalveolar lavage and she had an FEV1 of 75% predicted. At the age of 13 years, the patient was diagnosed with a latent pulmonary tuberculosis infection which was again treated with nine months of isoniazid monotherapy. At the age of 14 years, she was diagnosed with allergic bronchopulmonary aspergillosis, via elevated total IgE and Aspergillus-specific IgE. At this time, the patient had an FEV1 of 65% predicted.

**Figure 3:**
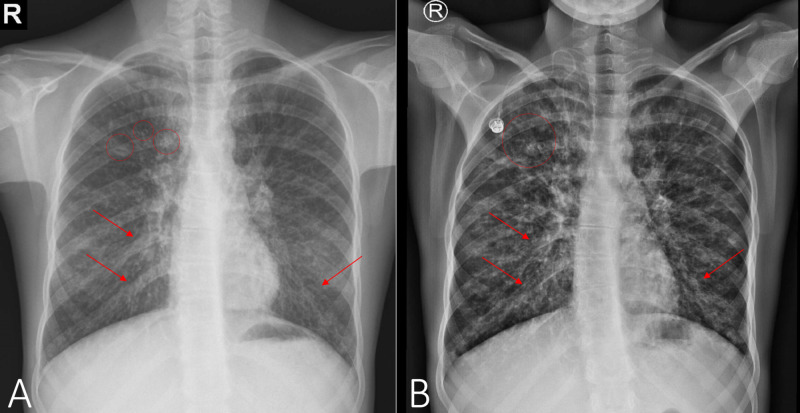
Chest radiography done in 2015 (A) and 2017 (B) showing worsening dilation of lower lobe bronchi (arrows) and area of signet-ring (circles) opacities in the right apical lung field

**Figure 4:**
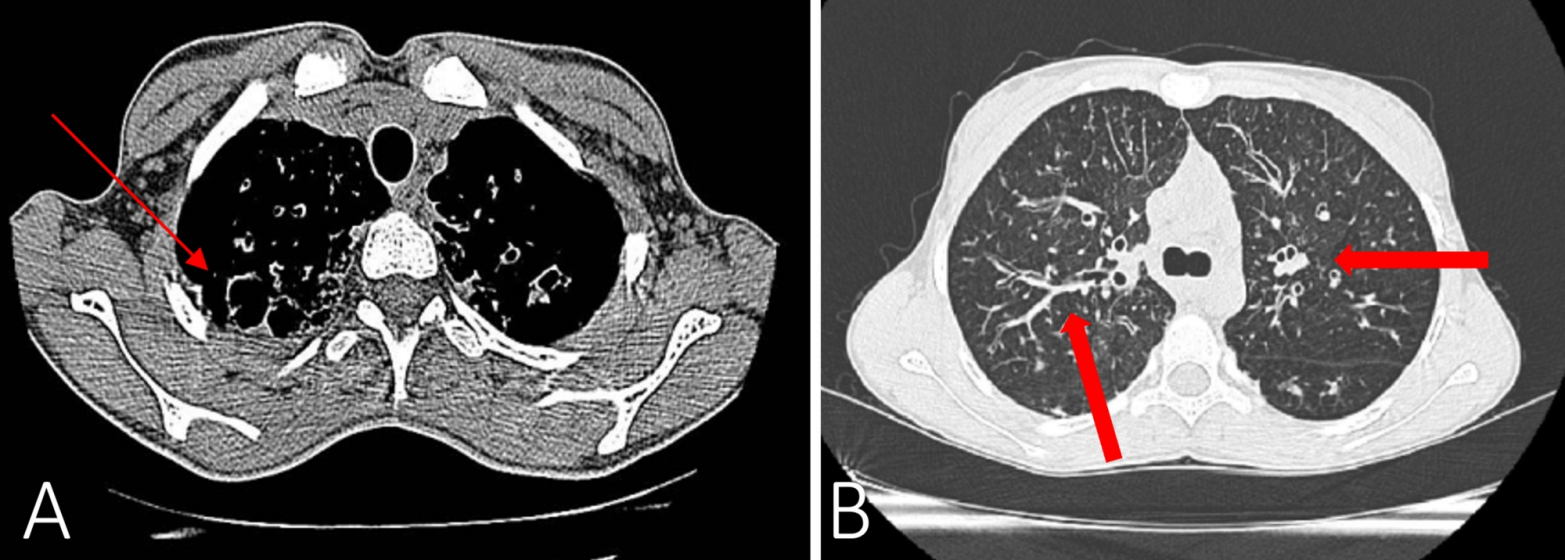
Chest CT of the apical lung field (A) showing the formation of cystic dilations on the right side (thin arrow) and of the middle lung field (B) showing signet-ring sign and cylindrical bronchiectasis (thick arrows)

At the age of 15 years (2015), colonization with BCC was noted, she had an FEV1 of 44% predicted. At this time, the patient showed first signs of the cutaneous vasculitis, in form of a palpable purpuric, nonvesicular maculopapular rash localized to the lower legs. The lower extremities were slightly swollen, painful, and as the episodes of the vasculitis continued to erupt, grey discoloration in the place of the rash began to set in and over the years became permanent. At the age of 18 years (2018), colonization with Achromobacter xylosoxidans and Candida parapsilosis was noted, she had an FEV1 of 24% during the exacerbation, which improved to 38% of predicted after treatment. The chest x-ray comparison from 2015 to 2017 is presented in Figure 3A and B, respectively. The CT scan done at this time showed cystic dilations in the right apex (Figure 4A) and severe bilateral middle lung field cylindrical bronchiectasis with signet-ring signs (Figure 4B).

Treatment was started with long-term oral prednisone. Colonization with Pseudomonas, BCC, MRSA, Achromobacter xylosoxidans, and Candida parapsilosis was treated with multiple courses of inhaled, IV and oral antibiotics (linezolid, ceftazidime, ofloxacin) and antifungals (fluconazole and voriconasole). In addition, the following was administered: azithromycin (three days a week for three months), tiotropium, an antihistamine, and periodic cough assist with antibiotic aerosol treatment. Starting from 2019, the patient’s nebulization with colistin and tobramycin was supplemented with nightly positive-pressure ventilation (PPV), but the ventilation was discontinued six months later due to slight hemoptysis that was predominantly present in the morning. Throughout the six months, the patient was on PPV, she experienced twp IECF, which required hospitalization. The two episodes of IECF, together with the extensive bronchiectasis, may have contributed to the appearance of the hemoptysis, which may have predisposed certain areas of the lung to barotrauma. After the PPV was discontinued, the hemoptysis disappeared and did not require any more treatment. 

## Discussion

CF is the most common monogenic lethal genetic disorder and the second most common hereditary metabolic disorder (after hemochromatosis) in the Caucasian population [3]. A mutation in the cystic fibrosis transmembrane conductance regulator (CFTR) gene leads to a defect in CFTR protein, which is an ATP-gated chloride channel [3]. Decreased chloride transport forms hyperviscous mucus, which blocks small passages in affected organs and perpetuates chronic inflammation and organ damage. When occurring in the lungs, this process of progressive pulmonary deterioration is the principal cause of morbidity and mortality in CF [3]. While pulmonary and pancreatic involvement is most frequent and therefore best studied, the cutaneous manifestations are frequently dismissed and underdiagnosed, despite being important markers of disease severity [2]. As much as 75% to 90% of patients who were diagnosed with purpura did not survive beyond two years [1,3,6]. Furthermore, if the cutaneous vasculitis becomes systemic, patients can expire as early as 10 days after rash onset [4]. BCC infection has also been linked to exacerbated inflammation and decline in lung function; it is one of the most common causes of morbidity and mortality in CF [7].

The cutaneous vasculitis seen in CF is classified as leukocytoclastic, although the exact cause is poorly understood and is presumed to be multifactorial. Implicated etiologies include adverse reactions to drugs (e.g., antibiotics and pancreatic enzymes), chronic inflammation of the airways leading to immune complex deposition, and a systemic response to bacterial colonization [8,9]. It is important to note that even though other bacterial infections, like Staphylococcus aureus and Pseudomonas aeruginosa have been associated with cutaneous vasculitis and were diagnosed in our patient, infection with BCC appeared before MRSA [10]. Therefore, BCC is the most likely causative agent of the cutaneous vasculitis. To our knowledge, only one other study in the literature currently exists, which explored this rare connection, concluding a high prevalence (16.7%) of cutaneous vasculitis in their cohort of patients with BCC colonization [5].

More recent studies may suggest a more complex mechanism - upregulation of CFTR was found to promote macrophage polarization into the anti-inflammatory M2 phenotype, which reduced the proinflammatory cytokine profile and vascular inflammation [11]. Furthermore, CFTR overexpression in pulmonary endothelial cells inhibits apoptosis and protects cells from oxidative stress, likely by inhibiting a signaling pathway [12]. Therefore, a lack of CFTR activity may promote an inflammatory state, which BCC colonization furthers presumably through cytokine secretion, as well as infecting and surviving intracellularly in dendritic cells (DCs) [7]. Infected DCs activate T-cells two times more often, suggesting non-antigen-specific activation [7]. How the interaction of BCC and decreased expression of CFTR ultimately culminates in cutaneous vasculitis is not fully elucidated and requires further research to better understand triggers and treatment.

Our patient’s manifestation of CF cutaneous vasculitis is consistent with other reports, namely, a palpable purpuric rash localized to the lower extremities, which appear in association with IECF [1,5,10]. Additionally, we noticed a clear correlation between the worsening of the vasculitis and episodes of IECF, but there is no evidence to support causation. Regarding respiratory function, the patient experienced a 10.25% predicted FEV1 decline per year since the diagnosis of the cutaneous vasculitis and colonization with BCC.

## Conclusions

A clear connection between cutaneous vasculitis exacerbations following pulmonary deterioration was noted in our patient; treatment of the infectious exacerbations of the lung disease with aggressive antibiotic association according to antibiograms was followed by improvement of cutaneous manifestation. We also underline that the clinical onset of the vasculitis was during the first lung infection with BCC. Our patient did not benefit from lung transplantation yet, and neither did she receive CFTR modulator therapy - additional research is required to determine if such interventions can cure the vasculitis.
